# Rising Intrahepatic Cholangiocarcinoma Rates in the United States Are Driving Liver Cancer Rates in Females

**DOI:** 10.1016/j.cgh.2025.12.013

**Published:** 2025-12-22

**Authors:** CHRISTIAN S. ALVAREZ, AIKA WOJT, ANDREA A. ALMEIDA, JEREMY S. MILLER, BARRY I. GRAUBARD, JESSICA L. PETRICK, KATHERINE A MCGLYNN

**Affiliations:** Division of Intramural Research, National Institute on Minority Health and Health Disparities, National Institutes of Health, Bethesda, Maryland; Division of Cancer Epidemiology and Genetics, National Cancer Institute, National Institutes of Health, Bethesda, Maryland; Murtha Cancer Center Research Program, Department of Surgery, Center for Prostate Disease Research, Uniformed Services University of the Health Sciences, Bethesda, Maryland; Henry M. Jackson Foundation for the Advancement of Military Medicine, Inc, Bethesda, Maryland.; Information Management Services, Inc, Calverton, Maryland; Division of Cancer Epidemiology and Genetics, National Cancer Institute, National Institutes of Health, Bethesda, Maryland; Slone Epidemiology Center, Boston University, Boston, Massachusetts; Division of Cancer Epidemiology and Genetics, National Cancer Institute, National Institutes of Health, Bethesda, Maryland

Intrahepatic cholangiocarcinoma (ICC), the second most commonly occurring liver cancer after hepatocellular carcinoma (HCC), is an increasingly important contributor to liver cancer incidence in the United States (US). Our previous studies have highlighted the contrasting trends in ICC and HCC incidence in recent decades.^1,2^ Although HCC incidence has declined, ICC incidence has risen. Recent analyses have reported that overall liver cancer rates continue to rise among females.^3^ To determine whether ICC underlies this increase, we examined long-term trends in ICC incidence and mortality in the US by sex and other demographic characteristics from 1992 to 2022.

ICC incidence^4^ and incidence-based mortality^5^ data were obtained from the Surveillance, Epidemiology, and End Results (SEER) program. Temporal trends were analyzed using the National Cancer Institute’s Joinpoint Regression Program (version 5.4.0.0). Further details are provided in the Supplementary Materials.

In 2022, the overall age-standardized ICC incidence rate (ASIR) was 2.08 per 100,000, and rates were similar in males (2.19) and females (2.00) (Table 1). Incidence increased with age, with the highest rates occurring among individuals aged 70 to 89 years (ASIR, 11.96). When stratified by race and/or ethnicity, no major differences were observed; the highest rates occurred among Hispanic individuals (ASIR, 2.44), followed by American Indian or Alaska Native individuals (AIAN) (ASIR, 2.27), and the lowest among Black individuals (ASIR, 1.92). By residence, incidence was slightly higher in urban areas (ASIR, 2.14) than in rural areas (ASIR, 1.69). Mortality rates mirrored incidence rates, with an overall age-standardized mortality rate of 1.73 per 100,000 (males, 1.88; females, 1.63).

Over the 31-year period, ICC incidence rose, with some fluctuations over time. There was an initial increase from 1992 to 1998 (estimated annual percentage change [EAPC], 3.50), a decline between 1999 and 2001 (EAPC, −7.53), then rates began a sustained increase starting in 2002 (EAPC, 4.70). Among males, incidence increased from 2003 to 2016 (EAPC, 5.22), then rose more modestly thereafter (EAPC, 1.89). Among females, rates increased continuously starting in 2003 (EAPC, 5.06). The parallelism test found that the trends by sex differed significantly (*P* = .003).

Most racial and/or ethnic groups experienced rising ICC incidence, although the increases started at different times. Although rates started rising in 1992 among White and Black individuals, the increases did not begin until the early 2000s among Hispanic and Asian or Pacific Islander (API) individuals. Patterns among AIAN individuals were harder to discern due to small case counts. Increases occurred among all age groups, and in both urban (EAPC, 4.79, since 2002) and rural (EAPC, 3.60, since 2003) areas.

Mortality trends closely mirrored incidence trends, with rates increasing steadily since 2003 (EAPC, 4.58). Increases were similar in males (EAPC, 4.32) and females (EAPC, 4.76), and across most racial, ethnic, and age groups.

Age-specific rates for the 1955 to 1964 birth cohort were the highest of all cohorts (Supplementary Figure 1). However, the oldest members of the 1955 to 1964 cohort (67 years old) had not yet attained the ages of peak ICC incidence (70–89 years) by 2022. Results from the age-period-cohort model indicated that both cohort (*P* < .001) and period effects (*P* < .001) contributed to the observed increase in ICC.

In notable contrast to ICC, HCC incidence rates are much higher among males (ASIR, 8.62) than females (ASIR, 2.80). HCC incidence has been declining since 2015 (EAPC, −3.52), with a more pronounced decrease among males (EAPC, −3.16 since 2012) than females (EAPC, −1.95 since 2013) (Supplementary Table 1). By 2022, the incidence of ICC had become nearly the same as the incidence of HCC among females (HCC:ICC ratio, 1.4), but not among males (HCC:ICC ratio, 3.9).

This analysis demonstrates that ICC rates have increased in the US, and in recent years, the increase has been more dramatic among females than males. In contrast, HCC rates have declined, albeit with slower decreases among females. As a result, the overall rise in liver cancer incidence among females largely reflects the increase in ICC incidence. As the rates of HCC and ICC are much more similar in females than males, the rising rates of ICC have had a disproportionate effect on the overall liver cancer rates of females.

The explanation for the opposing trends in HCC and ICC is not clear. The 2 types of liver cancer share a number of common risk factors (eg, hepatitis B virus, hepatitis C virus, metabolic dysfunction-associated steatotic liver disease), but most of the factors are more strongly associated with HCC than ICC.^6,7^ ICC is also associated with a number of bile duct conditions, most notably primary sclerosing cholangitis (PSC),^7^ and evidence suggests that the incidence and prevalence of PSC have been increasing.^8^ Although PSC is more common among males, PSC survival is more favorable among females, suggesting that females could be at higher long-term risk of ICC as a result. It is also possible that other, as yet unidentified, environmental risk factors for ICC may be driving risk. In addition, better diagnosis of ICC may be influencing the rising rates.^9^ Better diagnosis over time would be consistent with the calendar period effect on risk identified in the age-period-cohort analysis.

Although the differences in ICC rates by race and/or ethnicity are not as striking as those in HCC rates, disparities were evident, with Hispanic and AIAN populations experiencing the highest ICC incidence and mortality rates. These disparities may reflect unequal exposure to risk factors as well as barriers to early diagnosis and treatment.

These findings should be interpreted in light of several limitations. Case counts remain small for some racial and/or ethnic groups, particularly AIAN individuals, leading to less stable estimates. In addition, SEER lacks detailed individual-level data on risk factors.

In conclusion, ICC incidence and mortality have risen steadily in the US since the early 2000s, with the recent increases in risk being most pronounced among females. The increases in ICC have resulted in ICC now being the primary driver of rising liver cancer incidence among US females. These findings underscore the need for a better understanding of ICC risk factors to develop targeted prevention strategies.

## Supplementary Material

1

2

Supplementary Methods

Note: To access the supplementary material accompanying this article, visit the online version of *Clinical Gastroenterology and Hepatology* at www.cghjournal.org, and at https://doi.org/10.1016/j.cgh.2025.12.013.

## Figures and Tables

**Table 1.** Intrahepatic Cholangiocarcinoma, ASIR, ASMR and 95% CI in the United States: SEER Program 12 Registries, 1992 Through 2022

	1992			2022			EAPC, % Trend				Joinpoint, y Joinpoint			AAPC, 2000–2022	
	Cases	ASIR	95% CI	Cases	ASIR	95% CI	1	2	3	4	1	2	3	%	95% CI

Incidence	218	0.80	(0.70–0.92)	1008	2.08	(1.96–2.22)	3.50^a^	−7.53^a^	4.70^a^	-	1999	2002	-	3.13^a^	(2.83–3.52)
Sex															
Male	121	1.06	(0.88–1.28)	496	2.19	(2.00–2.40)	2.30^a^	−6.91^a^	5.22^a^	1.89	1999	2003	2017	2.29^a^	(1.93–2.78)
Female	97	0.63	(0.51–0.77)	512	2.00	(1.83–2.18)	8.22^a^	−1.50	5.06^a^	-	1996	2003	-	3.90^a^	(3.19–4.76)
Age at diagnosis^c^, *y*															
<50	26	0.12	(0.08–0.17)	88	0.35	(0.28–0.43)	2.37	27.04^a^	-	-	2020	-	-	3.86^a^	(2.22–5.00)
50–69	80	1.54	(1.22–1.92)	407	3.93	(3.55–4.33)	1.63	4.28^a^	-	-	2003	-	-	3.30^a^	(2.71–4.07)
70–89	105	4.67	(3.82–5.65)	476	11.96	(10.91–13.09)	3.79^a^	−11.14^a^	5.08^a^	-	1999	2002	-	3.03^a^	(2.64–3.64)
Race and/or ethnicity^b^															
White	135	0.66	(0.55–0.78)	560	2.03	(1.86–2.21)	5.06^a^	−12.64^a^	6.39^a^	3.98^a^	1999	2002	2014	3.38^a^	(3.00–3.84)
Black	11	0.58	(0.34–1.21)	75	1.92	(1.50–2.43)	3.39^a^	-	-	-	-	-	-	3.39^a^	(2.67–4.53)
Hispanic	34	1.33	(0.89–1.90)	192	2.44	(2.09–2.83)	0.04	4.34^a^	-	-	2005	-	-	2.45^a^	(1.60–3.43)
Asian or Pacific Islander	33	1.45	(0.98–2.06)	170	2.16	(1.85–2.52)	−1.06	2.45^a^	-	-	2003	-	-	1.15^a^	(0.37–1.97)
American Indian or Alaska Native	≤5	4.11	(1.27–9.29)	9	2.27	(1.01–4.37)	−0.68	-	-	-	-	-	-	−0.68	(−2.36 to 1.71)
Place of residence^d^															
Urban	179	0.80	(0.69–0.93)	927	2.14	(2.00–2.28)	3.78^a^	−7.45	4.79^a^	-	1999	2002	-	3.26^a^	(2.89–3.72)
Rural	26	0.67	(0.44–0.99)	79	1.69	(1.32–2.15)	−0.19	3.60^a^	-	-	2003	-	-	2.20^a^	(1.20–3.30)
Mortality	134	0.50	(0.42–0.59)	846	1.73	(1.61–1.85)	6.34^a^	−6.12 ^a^	4.58 ^a^	-	1999	2003	-	3.48^a^	(3.17–4.12)
Sex															
Male	72	0.65	(0.51–0.83)	421	1.88	(1.70–2.08)	4.67^a^	−10.18^a^	4.32 ^a^	-	2000	2003	-	2.86^a^	(2.54–3.52)
Female	62	0.40	(0.31–0.52)	425	1.63	(1.47–1.79)	14.97 ^a^	−1.92	4.76 ^a^	-	1996	2003	-	4.45^a^	(3.50–5.88)
Age at diagnosis^c^, *y*															
<50	10	0.05	(0.02–0.08)	41	0.16	(0.12–0.22)	2.22 ^a^	-	-	-	-	-	-	2.22 ^a^	(1.32–3.35)
50–69	48	0.94	(0.69–1.24)	325	3.12	(2.79–3.48)	3.52 ^a^	-	-	-	-	-	-	3.52 ^a^	(3.12–4.11)
70–89	71	3.17	(2.47–4.00)	441	11.09	(10.07–12.18)	6.60^a^	−7.60^a^	4.88 ^a^	-	1999	2003	-	3.51^a^	(3.07–4.22)
Race and/or ethnicity^b^															
White	76	0.37	(0.29–0.47)	475	1.67	(1.52–1.84)	34.33^a^	3.05	−9.28	5.15^a^	1994	2000	2003	4.89^a^	(3.98–5.71)
Black	7	0.47	(0.19–0.95)	66	1.71	(1.31–2.19)	3.10^a^	-	-	-	-	-	-	3.10^a^	(2.26–4.35)
Hispanic	24	0.94	(0.58–1.44)	157	2.00	(1.68–2.35)	3.07^a^	-	-	-	-	-	-	3.07^a^	(2.43–4.05)
Asian or Pacific Islander	23	1.05	(0.65–1.60)	139	1.76	(1.47–2.08)	−0.20	2.74^a^	-	-	2006	-	-	1.36^a^	(0.46–2.32)
American Indian or Alaska Native	≤5	3.55	(0.93–8.60)	7	1.59	(0.61–3.39)	−0.56	-	-	-	-	-	-	−0.56	(−2.27 to 1.86)
Residence^d^															
Urban	104	0.48	(0.39–0.58)	775	1.77	(1.64–1.90)	24.03^a^	2.79	−7.05	4.73^a^	1994	2000	2003	4.27^a^	(3.56–4.96)
Rural	20	0.51	(0.31–0.80)	69	1.39	(1.07–1.79)	2.54^a^	-	-	-	-	-	-	2.54^a^	(1.80–3.51)

AAPC, average annual percent change; ASIR, age-standardized incidence rate; ASMR, age-standardized mortality rate; CI, confidence interval; EAPC, annual percent change; SEER, Surveillance, Epidemiology and End Results.

a Denotes that the trend was statistically different from 0 at the alpha = .05 level.

b Cases may not sum to total due to unknown race and/or ethnicity categories (data not shown).

c Cases may not sum to total due to individuals older than 89 (data not shown).

d Cases may not sum to total due to unknown residence (data not shown).

## Data Availability

The data are available publicly with a data use agreement from the United States National Cancer Institute (https://seer.cancer.gov./data/).

## References

[1] Alvarez CS, Hepatology 2022;76:589–598. 10.1002/hep.32394 PMC9352816 35124828

[2] Petrick JL, Cancer 2020;126:3151–3155. 10.1002/cncr.32794 PMC7323860 32294255

[3] Siegel RL, CA Cancer J Clin 2025;75:10–45. 10.3322/caac.21871 PMC11745215 39817679

[4] Surveillance, Epidemiology, and End Results (SEER) Program. SEER*Stat Database: Incidence - SEER Research Data, 12 Registries, Nov 2024 Sub (1992–2022), National Cancer Institute, DCCPS, Surveillance Research Program, released April 2025, based on the November 2024 submission.

[5] Surveillance, Epidemiology, and End Results (SEER) Program. SEER*Stat Database: Incidence-Based Mortality - SEER Research Data, 12 Registries, Nov 2024 Sub (1992–2022), National Cancer Institute, DCCPS, Surveillance Research Program, released April 2025, based on the November 2024 submission.

[6] Clements O, J Hepatol 2020;72:95–103. 10.1016/j.jhep.2019.09.007 31536748

[7] Palmer WC, J Hepatol 2012;57:69–76. 10.1016/j.jhep.2012.02.022 PMC3804834 22420979

[8] Cooper J, Inflamm Bowel Dis 2024;30:2019–2026. 10.1093/ibd/izad276 PMC11532590 38052097

[9] Song Y, Eur J Med Res 2022;27:116. 10.1186/s40001-022-00741-9 PMC9277934 35820926

