# Supplementary figures and images for: Rising Intrahepatic Cholangiocarcinoma Rates in the United States Are Driving Liver Cancer Rates in Females
Source: Clin Gastroenterol Hepatol. Author manuscript; Available in PMC 2026 Apr 17. (PMC13086540; doi:10.1016/j.cgh.2025.12.013)

# ICC Incidence Rates by Birth Cohort and Age

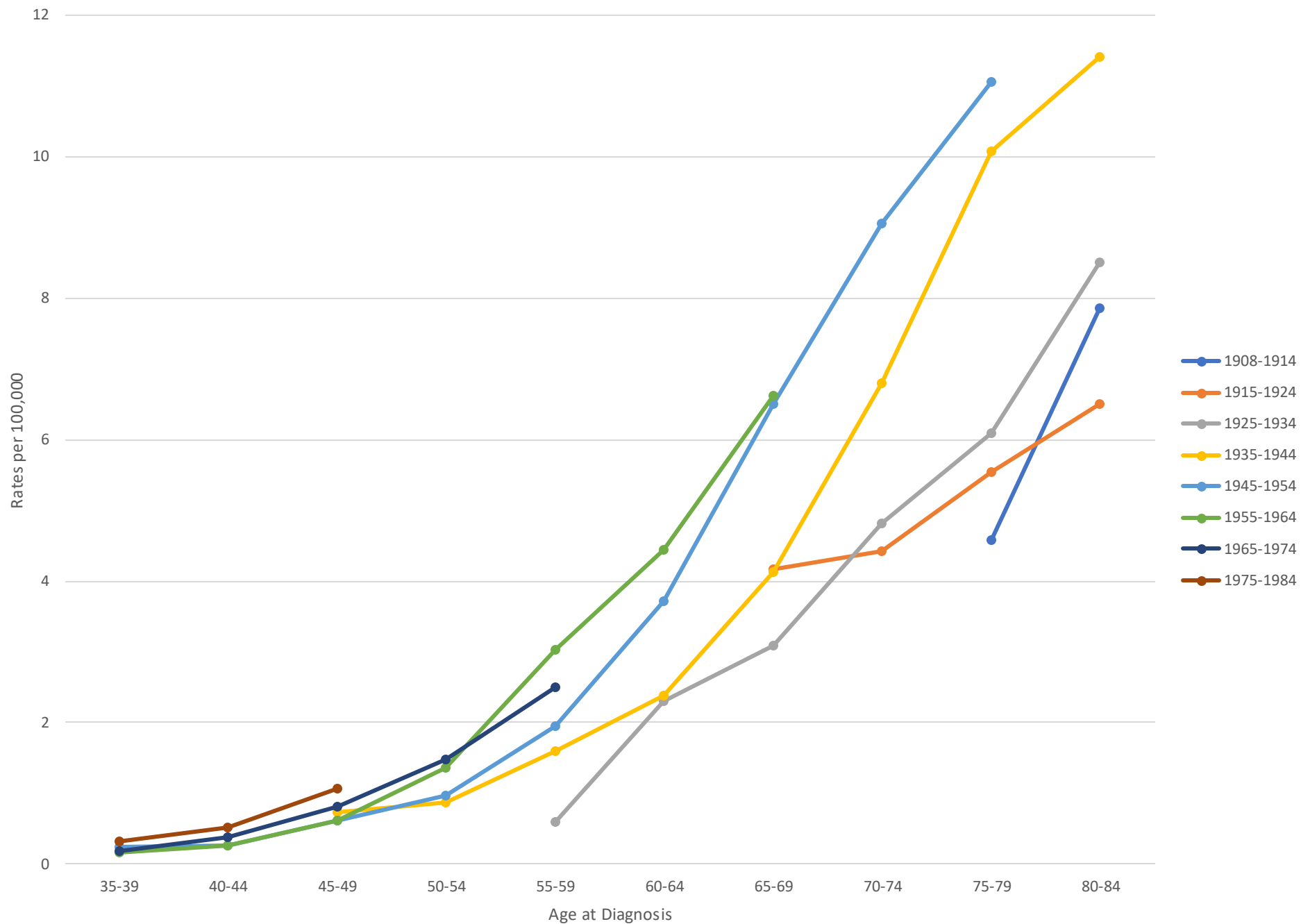

Supplement: 1 [file NIHMS2134040-supplement-1.pdf]
