# Supplementary material for: Rising Intrahepatic Cholangiocarcinoma Rates in the United States Are Driving Liver Cancer Rates in Females
Source: Clin Gastroenterol Hepatol. Author manuscript; Available in PMC 2026 Apr 17. (PMC13086540; doi:10.1016/j.cgh.2025.12.013)
Supplement: 2 [file NIHMS2134040-supplement-2.pdf]

Supplementary Table 1. Hepatocellular carcinoma ASIR, ASMR and 95% CI in the United States: SEER program 12 registries, 1992 through 2022

	1992			2022			EAPC, % Trend				Joinpoint years, Joinpoint			AAPC, 1992 to 2022	
	**Cases**	**ASIR**	**95%CI**	**Cases**	**ASIR**	**95%CI**	**1**	**2**	**3**	**4**	**1**	**2**	**3**	**%**	**95% CI**
**Incidence**	850	3.08	(2.88, 3.30)	2813	5.53	(5.33, 5.75)	4.67*	0.82	-3.52*	–	2009	2015	–	1.93*	(1.74, 2.15)
Sex															
Male	615	5.02	(4.62, 5.44)	2057	8.62	(8.25, 9.02)	4.34*	-3.16*	–	–	2012	–	–	1.78*	(1.57, 2.03)
Female	235	1.53	(1.34, 1.74)	756	2.80	(2.60, 3.01)	3.57*	-1.95*	–	–	2013	–	–	1.88*	(1.56, 2.28)
Age at diagnosis[c], y															
<50	118	0.53	(0.44, 0.64)	120	0.47	(0.39, 0.57)	9.34*	1.63	-4.74*	–	1998	2006	–	-0.37	(-1.11, 0.46)
50-69	372	7.15	(6.44, 7.92)	1416	13.35	(12.66, 14.08)	5.80*	1.34	-6.81*	–	2010	2015	–	1.98*	(1.78, 2.24)
70-89	351	15.45	(13.87, 17.16)	1218	30.24	(28.55, 32.00)	3.70*	0.35	–	–	2011	–	–	2.46*	(2.25, 2.70)
Race and/or ethnicity[b]															
White	388	1.89	(1.70, 2.08)	1155	3.84	(3.62, 4.08)	4.82*	-2.90*	–	–	2013			2.44*	(2.23, 2.69)
Black	70	3.85	(2.98, 4.89)	234	5.55	(4.84, 6.34)	5.45*	1.50	-8.24*	–	2007	2016	–	1.39*	(0.87, 2.13)
Hispanic	125	4.92	(4.05, 5.90)	782	9.33	(8.66, 10.04)	4.04*	-2.01*	–	–	2012	–	–	1.98*	(1.66, 2.46)
Asian or Pacific Islander	260	10.93	(9.59, 12.40)	570	7.15	(6.57, 7.77)	0.98*	-4.61*	–	–	2010	–	–	-1.30*	(-1.59, -0.97)
American Indian or Alaska Native	≤5	2.84	(0.65, 7.33)	48	10.87	(7.94, 14.57)	4.44*	-8.07	–	–	2017	–	–	2.24*	(0.94, 4.36)
Residence[d]															
Urban	720	3.17	(2.94, 3.41)	2533	5.56	(5.34, 5.79)	4.67*	0.36	-3.71*	–	2009	2015	–	1.79*	(1.60, 2.03)
Rural	67	1.82	(1.40, 2.32)	271	5.29	(4.66, 6.00)	6.06*	-0.48	–	–	2013	–	–	4.06*	(3.59, 4.66)
	**1992**			**2022**			**EAPC, % Trend**				**Joinpoint years Joinpoint**			**AAPC, 1992 to 2022**	
	**Deaths**	**ASMR**	**95%CI**	**Deaths**	**ASMR**	**95%CI**	**1**	**2**	**3**	**4**	**1**	**2**	**3**	**%**	**95% CI**
**Mortality**	489	1.78	(1.63, 1.95)	2764	5.45	(5.25, 5.66)	27.76*	3.47*	-0.82*	–	1994	2012	–	3.46*	(3.12, 3.97)
Sex															
Male	363	3.00	(2.70, 3.33)	2057	8.72	(8.34, 9.12)	25.23*	3.53*	-1.05*	–	1994	2012	–	3.28*	(2.87, 3.91)
Female	126	0.80	(0.67, 0.96)	707	2.59	(2.40, 2.79)	22.32*	2.47*	-0.20	–	1995	2013	–	3.48*	(2.98, 4.33)
Age at diagnosis[c], y															
<50	51	0.23	(0.17, 0.30)	73	0.29	(0.23, 0.36)	11.07*	-0.80	-6.56*	–	1998	2007	–	-1.53*	(-2.47, -0.44)
50-69	215	4.13	(3.59, 4.72)	1223	11.43	(10.79, 12.09)	25.69*	4.41*	-3.80*	–	1994	2013	–	3.15*	(2.58, 3.92)
70-89	218	9.62	(8.38, 10.98)	1388	34.45	(32.65, 36.32)	25.27*	3.30*	1.76	–	1994	2012	–	4.11	(3.67, 4.89)

Race and/or ethnicity[b]															
White	225	1.08	(0.94, 1.23)	1188	3.90	(3.68, 4.13)	30.11*	2.64	4.89	-1.18	1994	2003	2013	3.85*	(3.37, 4.49)
Black	44	2.36	(1.69, 3.18)	267	6.55	(5.77, 7.42)	4.71*	-1.29	–	–	2010	–	–	2.27*	(1.70, 3.00)
Hispanic	77	3.06	(2.38, 3.85)	714	8.93	(8.26, 9.63)	14.03*	2.80*	-0.21	–	1996	2012	–	3.20*	(2.40, 4.41)
Asian or Pacific Islander	140	6.08	(5.08, 7.21)	532	6.71	(6.15, 7.32)	23.97*	-0.15	-3.25*	–	1994	2011	–	0.14	(-0.31, 0.61)
American Indian or Alaska Native	≤5	0.63	(0.02, 3.36)	49	10.87	(7.97, 14.52)	275.21*	2.88	–	–	1994	–	–	12.15*	(2.79, 22.62)
Residence[d]															
Urban	420	1.86	(1.69, 2.05)	2508	5.54	(5.32, 5.76)	28.62*	3.37*	-1.17*	–	1994	2012	–	3.33*	(2.95, 3.91)
Rural	41	1.10	(0.78, 1.50)	246	4.67	(4.08, 5.32)	5.46*	1.02	–	–	2014	–	–	4.25*	(3.80, 4.86)

Abbreviations: 95% CI, 95% confidence interval; AAPC, average annual percent change; EAPC, estimated annual percent change; ASIR, age-standardized incidence rate; ASMR, age-standardized mortality rate; SEER, Surveillance, Epidemiology and End Results.

[a]Asterisk denotes that the trend was statistically different from 0 at the alpha =.05 level.

[b]Cases may not sum to total due to unknown race and/or ethnicity categories (data not shown).

[c]Cases may not sum to total due to individuals older than 89 (data not shown).

[d]Cases may not sum to total due to unknown residence (data not shown).

HCC were classified using the International Classification of Disease for Oncology, Third Edition (ICD-O-3), with topography code C22. HCC was identified by morphology codes 8170-8175
