# Supplementary material for: Rising Intrahepatic Cholangiocarcinoma Rates in the United States Are Driving Liver Cancer Rates in Females
Source: Clin Gastroenterol Hepatol. Author manuscript; Available in PMC 2026 Apr 17. (PMC13086540; doi:10.1016/j.cgh.2025.12.013)
Supplement: Supplementary Methods [file NIHMS2134040-supplement-Supplementary_Methods.docx]

**DETAILED METHODS:**

The data were drawn from the Surveillance, Epidemiology, and End Results (SEER) program 12 registries (1992-2022), which represents 12% of the U.S. population. ICC was identified using ICD-O-3 topography code C22 and morphology codes 8032-8033, 8041, 8050, 8070-8071, 8140-8141, 8160, 8260, 8480-8481, 8490, and 8560. Age-standardized rates (per 100,000 person-years) were calculated using the 2000 U.S. standard population and stratified by sex, race and/or ethnicity (non-Hispanic White [White], non-Hispanic Black [Black], Hispanic, non-Hispanic Asian/Pacific Islander [API], and non-Hispanic American Indian/Alaska Native [AIAN] [purchased/referred care delivery area only]), place of residence (urban, rural), and age at diagnosis (<50, 50-69, ≥70 years). Data on HCC (ICD-0-3 topography code C22, morphology codes 8170-8175) were also identified in order to compare trends with ICC.

Estimated annual percent change (EAPC) and average annual percent change (AAPC) with corresponding 95% confidence intervals were computed using the National Cancer Institute’s (NCI) Joinpoint Regression Program, version 5.4.0 (https://surveillance.cancer.gov/joinpoint/). A pairwise test of parallelism, using the Joinpoint program, was conducted to compare ICC trends by sex. To visualize trends by birth cohort, age-specific ICC incidence rates by birth year were plotted, and age-period-cohort (APC) models were applied to evaluate the independent effects of age, calendar period, and birth cohort, using Wald chi-square tests implemented in the NCI’s APC Analysis Web Tool (analysistools.cancer.gov/apc/). All p values were two-sided and a p value ≤ 0.05 was considered statistically significant.
